# Comparison of 3 γ-probes for simultaneous iodine-125-seed and technetium-99m breast cancer surgery: NEMA standard characterisation with extended processing

**DOI:** 10.1186/s40658-020-00299-7

**Published:** 2020-06-05

**Authors:** Christopher Hoog, Pierre-Malick Koulibaly, Catherine Dejean, Tom Desdoits, Olivier Humbert, Emmanuel Barranger, Jacques Darcourt

**Affiliations:** 1 Department of diagnostic radiology and nuclear medicine, Antoine Lacassagne Comprehensive Cancer Center, Université Nice-Côte d’Azur, 33 Avenue de Valombrose, 06189 Nice, France; 2 Department of worker radiation protection, Antoine Lacassagne Comprehensive Cancer Center, Université Nice-Côte d’Azur, 33 Avenue de Valombrose, 06189 Nice, France; 3 Department of oncological, gynaecological and breast surgery, Antoine Lacassagne Comprehensive Cancer Center, Université Nice-Côte d’Azur, 33 Avenue de Valombrose, 06189 Nice, France

**Keywords:** Breast surgery, γ-Probes, Radioactive seed localisation, NEMA testing

## Abstract

**Purpose:**

Iodine-125 (^125^I) seeds can be used as landmarks to locate non-palpable breast lesions instead of implanting metal wires. This relatively new technique requires a nuclear probe usually used for technetium-99m (^99m^Tc) sentinel node detection. This study aimed to compare the performances of different probes and valid the feasibility of this technique, especially in the case of simultaneous ^125^I-seed and ^99m^Tc breast cancer surgery.

**Methods:**

Three probes with different features (SOE-3211, SOE-3214 and GammaSUP-II) were characterised according to the NEMA NU3-2004 standards for a ^99m^Tc source and a ^125^I-seed. Several tests such as sensitivity, linearity or spatial resolution allowed an objective comparison of their performances. NEMA testing was extended to work on signals discrimination in case of simultaneous detection of two different sources (innovative figure of merit “Shift Index”) and to assess the ^99m^Tc scatter fraction, a useful parameter for the improvement of the probes in terms of detector materials and electronic system.

**Results:**

Although the GammaSUP-II probe saturated at a lower activity (1.6 MBq at 10 mm depth), it allowed better sensitivity and spatial resolution at the different NEMA tests performed with the ^99m^Tc source (7865 cps/MBq and 15 mm FWHM at 10 mm depth). With the ^125^I-seed, the GammaSUP-II was the most sensitive probe (3106 cps/MBq at 10 mm depth) and the SOE-3211 probe had the best spatial resolution (FWHM 20 mm at 10 mm depth). The SOE-3214 probe was more efficient on discriminating ^125^I from ^99m^Tc in case of simultaneous detection. The SOE probes were more efficient concerning ^99m^Tc scatter fraction assessments. The SOE-3211 probe, with overall polyvalent performances, seemed to be an interesting trade-off for detection of both ^125^I and ^99m^Tc.

**Conclusion:**

The three probes showed heterogeneous performances but were all suitable for simultaneous ^99m^Tc sentinel node and ^125^I-seed detection. This study provides an objective and innovative methodology to compare probes performances and then choose the best trade-off regarding their expected use.

## Introduction

For the excision of non-palpable breast lesions, the surgeon needs a landmark previously placed under radiological or ultrasound imaging control. Guided surgery is an evolving field [1], whether it concerns fluorescence [2], ferromagnetic detection [3], radiofrequency [4] or radioactivity detected by γ-camera [5]. But in France, the gold standard technique remains implanting a metal wire from the lesion to the skin. The Antoine Lacassagne Centre (Nice, France) is the first French hospital authorised to offer an alternative solution with an iodine-125-seed (^125^I-seed) implanted into the tumour [6]. The two techniques are currently compared within the randomised prospective clinical trial “IODINE BREAST” (NCT02759133). The literature already tips the scales in favour of the ^125^I-seed technique [7]. Indeed, using the metal wire leads to more important margins and involves some discomfort for the patient. The iodine technique seems to be an answer to these issues with a lower rate of repeated surgery and a lower psychological impact [8, 9]. However, specific nuclear probes are needed to detect the seed into the breast.

**Fig. 1:**
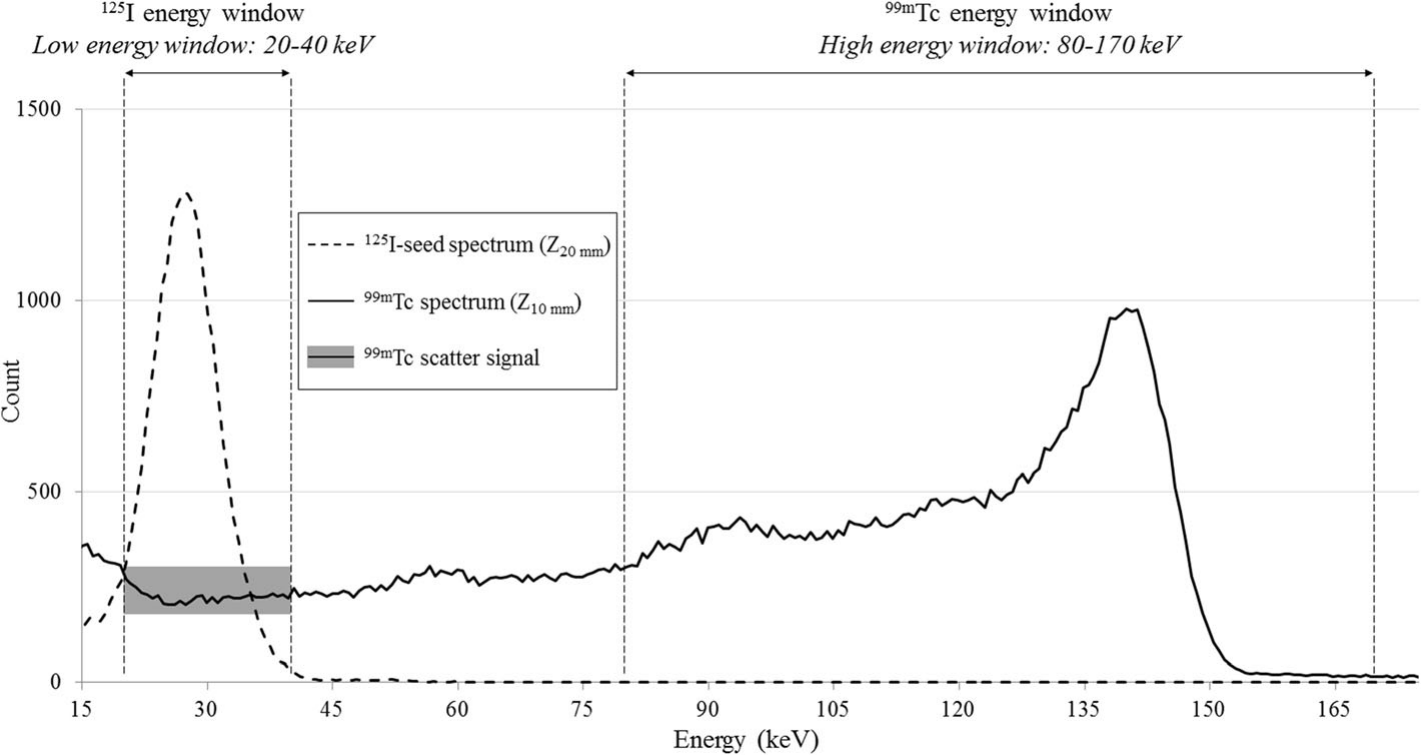
Illustration of the EURORAD low (20–40 keV) and high (80–170 keV) energy windows onto the energy spectra of a ^99m^Tc source and a ^125^I-seed in a scatter medium performed with the SOE-3214 probe. The sources depths (Z) were considered from the in vitro clinical simulations. These spectra were acquired over 30 s and are presented for 1 MBq activity. The ^99m^Tc scatter signal corresponds to the ^99m^Tc signal recorded in the low energy window

In the context of breast tumour surgery, it is common to inject technetium-99m (^99m^Tc)-nanocolloids around the areola to find and remove the sentinel node, using nuclear probes as well. The ^125^I and ^99m^Tc radionuclides emit photons of 27 and 140 keV respectively; thus, ^99m^Tc scatter signal can impact on the detection of the ^125^I-seed (Fig. 1).

The main purpose of our study was the characterisation of three different gamma probes for both sources of ^99m^Tc and ^125^I according to the NEMA NU3-2004 international standards [10]. We aimed to go further exploiting data from two NEMA tests leading to two additional indexes:

- The “Shift Index” was defined as an innovative figure of merit of signals discrimination in case of simultaneous detection of two different sources.
- The ^99m^Tc scatter fraction was calculated according to the source activity and depending on the spatial positioning of the probe. This parameter is useful to improve the probes, especially in terms of detector materials and electronic system.

The probes performances were compared in order to determine if one of them was more suitable to this technique, especially in terms of simultaneous detection of ^125^I-seed and ^99m^Tc sources.

## Material and methods

### Probes (Table 1)

Three gamma probes (without external collimator) from two companies with a quality control up to date were considered for this study: SOE-3211 and SOE-3214 (EURORAD, Strasbourg, France) and GammaSUP-II (CLERAD, Clermont-Ferrand, France).

**Table 1:** Probes’ features

Company	Model	Type	Detector	Detector dimensions (mm^3^)	Entrance layer	Energy range (keV)
EURORAD	SOE-3211	Nuclear	CdTe	Cubic ≈ 5 × 5 × 3 (69)	Stainless-steel	^125^I: 20–40 ^99m^Tc: 80–170
EURORAD	SOE-3214	Opto-Nuclear	CdTe	Orthocylindrical ≈ *Ø* 5 × 3 (57)	Resin	^125^I: 20–40 ^99m^Tc: 80–170
CLERAD	GammaSUP-II	Nuclear	CSI[Tl]	Orthocylindrical *Ø* 7 × 7 (269)	Stainless-steel	^125^I: 20–40 ^99m^Tc: 120–170

*CdTe* cadmium telluride, *CSI[Tl]* thallium-doped caesium iodide, ^*125*^*I* iodine-125, ^*99m*^*Tc* technetium-99m

*SOE-3211 from EURORAD* is a bent nuclear probe equipped with a cadmium telluride (CdTe) semiconductor detector designed to detect photons in the range 20-170 keV. Thus, it is dedicated to the detection of ^125^I (27 keV) and ^99m^Tc (140 keV). The head of the probe is 11 mm diameter with a stainless-steel entrance layer.

*SOE-3214 from EURORAD* is a straight opto-nuclear probe with the same detection features as the SOE-3211 probe. In addition to detect ^125^I and ^99m^Tc signals, two optical fibres allow infracyanine green fluorescence for sentinel nodes detection. The head of the probe is 12 mm diameter with a resin entrance layer.

*GammaSUP-II from CLERAD* is a straight nuclear probe equipped with a thallium-doped caesium iodide [CsI(Tl)] scintillator coupled with a photomultiplier for the detection of photons in the energy range 20-400 keV. Thus, this probe can detect photons of low and high energies. The head of the probe is 12 mm diameter with a stainless-steel entrance layer.

For every NEMA standards testing, count rates were recorded in the clinical energy windows corresponding to the source: low energy window (20–40 keV for EURORAD and CLERAD) for the ^125^I-seed and high energy window (80–170 keV for EURORAD and 120–170 keV for CLERAD) for the ^99m^Tc source. For the extended NEMA testing, (clinical simulations and ^99m^Tc scatter fraction), ^99m^Tc scatter signal was measured in the low energy window while performing the spatial resolution and count rate capability tests (Table 2, Fig. 1).

**Table 2:** Exploitation of the measurements while performing the spatial resolution and count rate capability tests

		Low energy window				High energy window				
^99m^Tc	Spatial resolution		2	3		1		3		
	Count rate capability				4	1			4	
^125^I	Spatial resolution	1	2							
	Count rate capability	1								

1: NEMA 2: Clinical simulations 3: Spatial scatter fraction 4: Activity scatter fraction

### NEMA testing (Fig. 2)

Among every NEMA tests, we chose the seven most relevant ones for our study which were practically feasible: sensitivity in air, sensitivity in a scatter medium, sensitivity through side shielding in air, sensitivity to scatter, spatial resolution in a scatter medium, count rate capability in a scatter medium and side and back shielding. Each test was performed according to the NEMA NU3-2004 standards [11] with some adaptations for the in vitro clinical simulations and ^99m^Tc scatter fraction assessments.

**Fig. 2:**
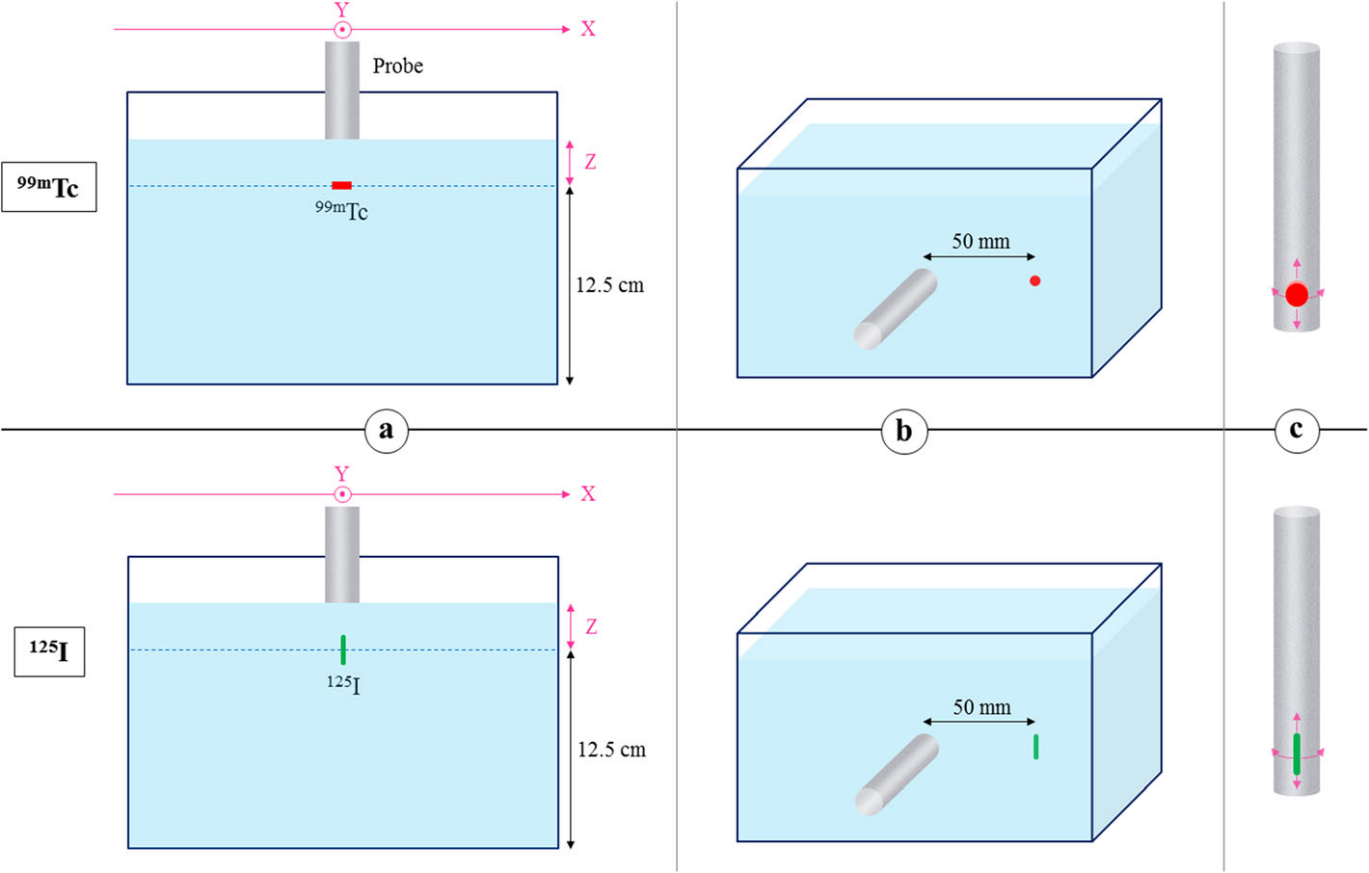
Schematic representation of the count rate capability (***Z*** = 10 mm), sensitivity (***Z*** = 10, 30 and 40 mm) and spatial resolution (***Z*** = 10, 20 and 30 mm) in a scatter medium (**a**), sensitivity to scatter (**b**) and side and back shielding (**c**) tests with a ^99m^Tc source and a ^**125**^I-seed. For the sensitivity in air (***Z*** = 0, 10, 30 and 50 mm) and sensitivity through side shielding in air assessments, **a** and **b** setups were respectively used without water. The probe was slided through the ***X*** axis for the spatial resolution test; it was fixed at ***X*** = 0 mm for every other tests

^99m^Tc sources were prepared by pouring liquid drops in a plastic capsule filled with paper and clogged with wax to be sealed. The capsule was a cylinder of 2.5 ± 0.5 mm diameter by 1.5 ± 0.5 mm height and was assumed to be punctual (volume < 14 μL). The activity was measured using a MEDI 404 (MEDISYSTEM, Guyancourt, France) dose calibrator with an accuracy of less than 5%.

^125^I sources IsoSeed® I25.S06 were provided by Eckert & Ziegler BEBIG as cylinder seeds of 0.8 mm diameter × 4.5 mm height. We considered the activity from the calibration certificate taking into account the radioactive decay.

The measurements were performed using a water tank 36.5 cm long × 21.4 cm large × 16.5 cm height, filled or not with water according to the tests and a Fisso arm to ensure a precise, stable and reproducible probe positioning.

#### Sensitivity in air

The source was fixed at the centre of the air tank at 12.5 cm height. The probe was aligned with the source along the vertical axis. Measurements were performed with the tip of the probe at 0 (contact), 10, 30 and 50 mm above the source.

#### Sensitivity in a scatter medium

The source was fixed at the centre of the water tank at 12.5 cm height. The head of the probe was aligned with the source along the vertical axis. Measurements were performed with the tip of the probe touching the water surface at 10, 30 and 40 mm above the source.

#### Sensitivity through side shielding in air

The probe was positioned at the centre of the wall of the air tank and aligned along the horizontal axis. The source was fixed 50 mm further on the same wall tank and at the same height.

#### Sensitivity to scatter

The setup was the same as the one used for the sensitivity through side shielding in air test at the exception the tank was filled with water. For a same probe and source, if the ratio between the count rate without and with scatter medium exceeded 10%, the sensitivity to scatter value was corrected by subtracting the sensitivity through side shielding in air value.

#### Spatial resolution in a scatter medium

From the setup used for the sensitivity in a scatter medium test, several measurements were performed through the *X* axis from − 50 to + 50 mm with the probe aligned according to the vertical axis at 10, 20 and 30 mm above the source. The measurement steps were adapted according to the distance from the *X* axis origin: 2.5 mm steps for 0 < |*X*| < 5 mm; 5 mm steps for 5 < |*X*| < 20 mm; 10 mm steps for 20 < |*X*| < 50 mm. The two measurements for the same distance from *X* axis origin (on *X* negative and *X* positive) were averaged in order to obtain symmetrical curves. Spatial resolution was expressed as the full width at half maximum (FWHM) and the full width at tenth maximum (FWTM) using linear interpolation around the 50% and 10% of the maximum respectively.

#### Count rate capability in a scatter medium

The source was fixed at the centre of the water tank at 12.5 cm height. The probe was aligned with the source along the vertical axis. Measurements were performed with the tip of the probe touching the water surface, 10 mm above the source. For ^99m^Tc, measurements were performed over 48 h to obtain count rate values for an activity ranging from 0.1 to 7 MBq. For ^125^I, we used different seeds to obtain count rate values for an activity ranging from 1 to 10 MBq. The count rate capability corresponded to the activity value above which the loss of count rate linearity exceeded 20%.

#### Side and back shielding

The source was slowly moved around the probe’s head surface and in contact with it. It was ensured the source did not shine directly the detector from the tip of the probe. The highest count rate and the source position associated were recorded.

From this test and the sensitivity in air, the shielding effectiveness (SE) was calculated (Eq. 1).

1 $$ \mathrm{SE}=\frac{\ \mathrm{Sensitivity}\ \mathrm{in}\ {\mathrm{air}}_{\mathrm{contact}}-\mathrm{Side}\ \mathrm{and}\ \mathrm{back}\ \mathrm{shielding}}{\mathrm{Sensitivity}\ \mathrm{in}\ {\mathrm{air}}_{\mathrm{contact}}}\times 100 $$

For the sensitivity, spatial resolution and count rate capability tests (in air and scatter medium), the probe position was adjusted until an equivalent count rate at ± 15 mm on the *X* and *Y* axis was measured. This methodology ensured to be at the maximum count rate at the origin of the *XY* plane.

For every test, we used activities in agreement with the linear response of the probes obtained with the count rate capability test.

The results of sensitivity and shielding tests were reported as count rate per unit of radioactivity (cps/MBq). The results of spatial resolution were reported as millimetre (mm). Count rate capability values were reported as megabecquerel (MBq).

### Extended NEMA testing

#### Shift Index

**Fig. 3:**
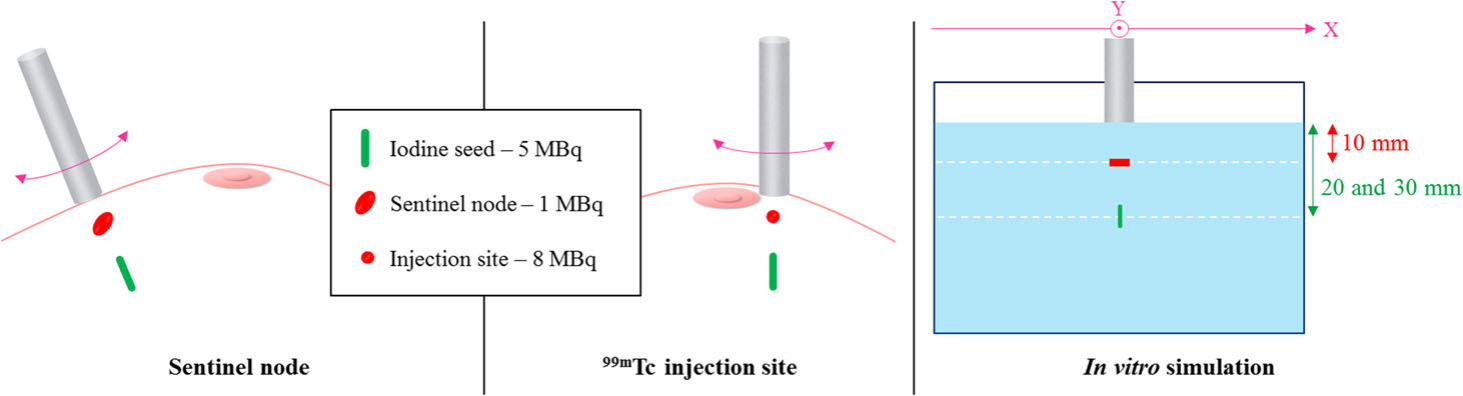
In vitro simulations of two unfavourable clinical configurations

**Fig. 4:**
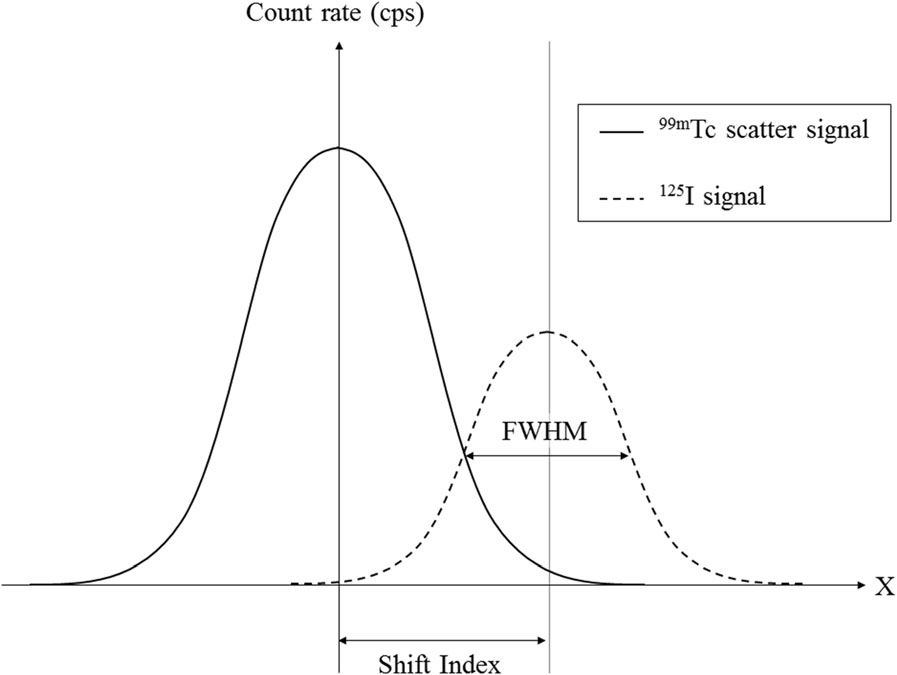
Illustration of the “Shift Index” figure of merit

For simultaneous ^125^I-seed and ^99m^Tc breast cancer surgery, probes have to detect and discriminate the signals from both radionuclides. Two unfavourable clinical situations were simulated in vitro: a ^125^I-seed implanted below a ^99m^Tc injection site and a ^125^I-seed implanted below a ^99m^Tc sentinel node. For both configurations, we considered the ^99m^Tc source at 10 mm depth and the ^125^I-seed at 20 and 30 mm depth (Fig. 3). We compared the spatial resolution profiles measured at these depths in the low energy window for both sources (^125^I signal vs ^99m^Tc scatter signal). The count rates were multiplied by clinical activities without taking into account probes linearity issues: 1, 5 and 8 MBq for the sentinel node, the ^125^I-seed and the injection site, respectively. When the ^99m^Tc scatter signal was higher than the ^125^I signal, we defined a Shift Index as the minimum distance along the *X* axis needed to discriminate the two sources. The Shift Index was calculated as the limit of the “visible” FWHM of the ^125^I signal, while moving the sources apart from each other (Fig. 4).

#### ^99m^Tc scatter fraction

The ^99m^Tc scatter fraction, which only involved ^99m^Tc sources, is of major interest for the manufacturers. Indeed, they use to rely on its assessment to improve the structure of the crystal detector and the electronic system to reach the lowest scatter fraction achievable.

Compton interactions in the scatter medium or in the detection system, and electronic noise, are at the origin of the ^99m^Tc scatter signal. The scatter fraction index (SF) was defined as the ratio between the signal measured in the low energy window (^99m^Tc scatter) over the signal measured in the high energy window (photopeak).

Two SF were calculated, the first one as a function of activity (Activity SF) (Eq. 2) and the second one depending on the spatial positioning of the probe (Spatial SF) (Eq. 3) while performing the count rate capability and spatial resolution NEMA tests, respectively.

2 $$ \mathrm{Count}\ \mathrm{rate}\ \mathrm{capability}\ \mathrm{test}\to \mathrm{Activity}\ \mathrm{SF}\left(A,{X}_{0\ \mathrm{mm}},{Z}_{10\ \mathrm{mm}}\right)=\frac{\ {\mathrm{Count}\ \mathrm{rate}}_{\mathrm{low}\ \mathrm{energy}\ \mathrm{window}}(A)}{{\mathrm{Count}\ \mathrm{rate}}_{\mathrm{high}\ \mathrm{energy}\ \mathrm{window}}(A)}\times 100 $$

3 $$ \mathrm{Spatial}\ \mathrm{resolution}\ \mathrm{test}\to \mathrm{Spatial}\ \mathrm{SF}\left({A}_0,X,{Z}_{10\ \mathrm{mm}},{Z}_{20\ \mathrm{mm}},{Z}_{30\ \mathrm{mm}}\right)=\frac{\ {\mathrm{Count}\ \mathrm{rate}}_{\mathrm{low}\ \mathrm{energy}\ \mathrm{window}}\left(X,Z\right)}{{\mathrm{Count}\ \mathrm{rate}}_{\mathrm{high}\ \mathrm{energy}\ \mathrm{window}}\left(X,Z\right)}\times 100 $$

## Results

The three probes worked properly and without any problem during the tests. The GammaSUP-II system needed a fifteen minutes warm-up before ensuring a stable measurement.

### NEMA testing

#### Sensitivity (Table 3)

Sensitivity results are presented with their standard deviations but assuming a 0.5 mm spatial error positioning, a 15% error has to be added in quadrature [11].

**Table 3:** Sensitivity in air, sensitivity in a scatter medium and sensitivity to scatter corrected from sensitivity through side shielding in air for the three probes

		Sensitivity in air (cps/MBq)				Sensitivity in a scatter medium—water (cps/MBq)			Sensitivity to scatter corrected (cps/MBq)
	Source depth	0 mm	10 mm	30 mm	50 mm	10 mm	30 mm	40 mm	-
^99m^Tc	SOE-3211	14386 ± 99	2581 ± 29	564 ± 8	274 ± 6	2774 ± 22	422 ± 8	283 ± 5	13.4 ± 2.8
	SOE-3214	9688 ± 54	2143 ± 33	484 ± 7	183 ± 6	2402 ± 23	347 ± 9	228 ± 4	10.2 ± 2.4
	GammaSUP-II	28016 ± 100	8063 ± 33	1521 ± 17	606 ± 9	7865 ± 30	935 ± 14	571 ± 9	11.1 ± 4.3
^125^I	SOE-3211	3574 ± 9	603 ± 4	98 ± 2	40 ± 1	459 ± 3	64 ± 1	31 ± 1	0.8 ± 0.3
	SOE-3214	6245 ± 18	1500 ± 8	267 ± 2	104 ± 2	1653 ± 9	210 ± 2	109 ± 2	4.9 ± 0.6
	GammaSUP-II	12182 ± 11	3177 ± 10	403 ± 2	156 ± 25	3106 ± 10	333 ± 3	138 ± 2	6.5 ± 0.8

The GammaSUP-II probe was more sensitive than the two other probes in air and scatter medium for both ^99m^Tc and ^125^I sources. Regarding the SOE-3211 and SOE-3214 probes, the first one was more sensitive for the ^99m^Tc, whereas the second one was more sensitive for ^125^I. For example, at 10 mm depth in water, the sensitivities for the SOE-3211, SOE-3214 and GammaSUP-II were, respectively, 2774, 2402 and 7865 cps/MBq with the ^99m^Tc source and 459, 1653 and 3106 cps/MBq with the ^125^I-seed.

The sensitivity to scatter corrected from sensitivity through side shielding in air was equivalent for the 3 probes with the ^99m^Tc source (between 10.2 and 13.4 cps/MBq). With the ^125^I-seed, the SOE-3211 probe showed better results (0.8 cps/MBq), while the SOE-3214 and GammaSUP-II probes seemed more sensitive to the scatter with 4.9 and 6.5 cps/MBq, respectively.

#### Spatial resolution in a scatter medium (Table 4, Fig. 5)

With the ^99m^Tc source, the GammaSUP-II probe allowed a better spatial resolution, while the SOE probes showed equivalent results (except for the FWHM at 10 mm depth). With the ^125^I-seed, the SOE-3211 probe allowed a better spatial resolution while the SOE-3214 and the GammaSUP-II probe had equivalent results. For example, at 20 mm depth, the FWHM for the SOE-3211, SOE-3214 and GammaSUP-II probes were respectively 28, 29 and 25 mm with the ^99m^Tc source and 30, 34 and 34 mm with the ^125^I-seed.

**Table 4:** Spatial resolution in a scatter medium for the three probes

		FWHM (mm)			FWTM (mm)		
	Source depth	10 mm	20 mm	30 mm	10 mm	20 mm	30 mm
^99m^Tc	SOE-3211	17	28	40	37	62	91
	SOE-3214	20	29	42	38	59	91
	GammaSUP-II	15	25	35	30	57	77
^125^I	SOE-3211	20	30	40	35	56	76
	SOE-3214	24	34	45	41	62	84
	GammaSUP-II	25	34	48	40	62	86

*FWHM* full width at half maximum, *FWTM* full width at tenth maximum

**Fig. 5:**
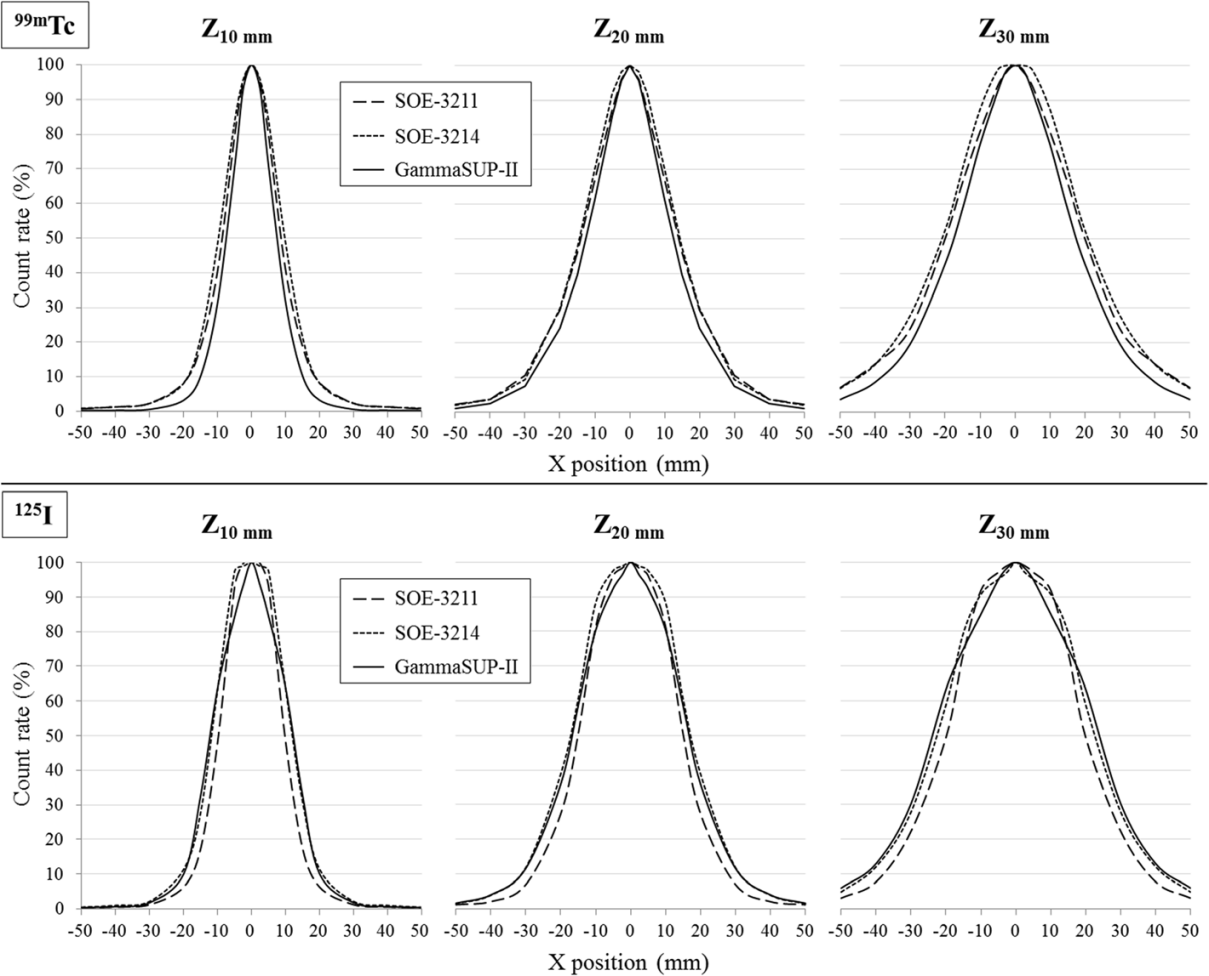
Spatial resolution profiles of the three probes for ^99m^Tc and ^125^I sources at 10, 20 and 30 mm depths

#### Count rate capability in a scatter medium (Fig. 6)

The GammaSUP-II probe saturated above 1.6 MBq with a ^99m^Tc source at 10 mm depth, while the SOE-3211 and SOE-3214 probes saturated above 7.4 and 5.6 MBq, respectively. With the ^125^I-seed, no saturation was observed, indeed the count rate remained linear from 1 to 10 MBq for the three probes.

**Fig. 6:**
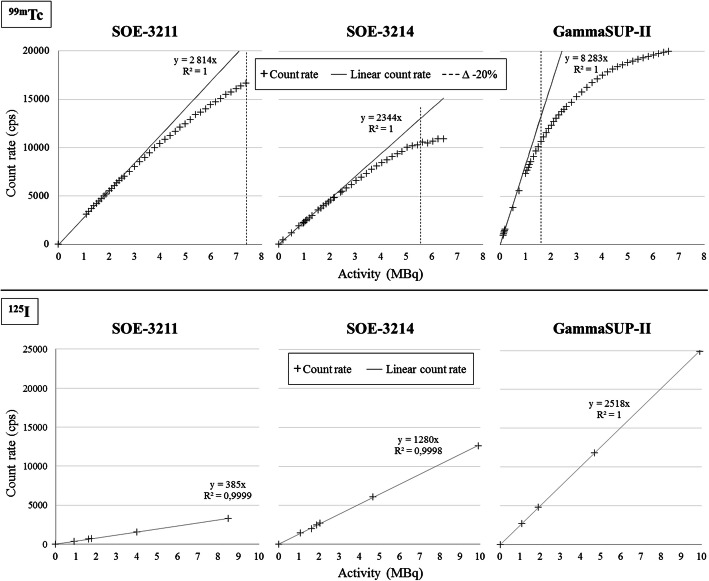
Count rate capability of the three probes for ^99m^Tc and ^125^I sources at 10-mm depth

#### Side and back shielding

The side and back shielding of the three probes seemed to be efficient with results upper than 98% for ^99m^Tc and equal to 100% for ^125^I.

### Extended NEMA testing

#### Shift index (Table 5, Fig. 7)

##### Injection site simulation:

For the three probes, the ^99m^Tc scatter signal at 10 mm depth was higher than the ^125^I-seed signal at 20 and 30 mm depth. The SOE-3214 probe allowed a better discrimination of the two sources. This probe led to the smallest differences between ^125^I and ^99m^Tc scatter signal intensities and the lowest Shift Index values (26 and 37 mm with the ^125^I-seed at 20 and 30 mm depth, respectively).

**Table 5:** Shift Index (mm) from clinical simulations for the three probes

	^99m^Tc Injection site (10 mm depth)		^99m^Tc Sentinel node (10 mm depth)	
^125^I-seed depth	20 mm	30 mm	20 mm	30 mm
SOE-3211	32	46	N/A	30
SOE-3214	26	37	N/A	N/A
GammaSUP-II	33	48	N/A	35

*N/A* not applicable

**Fig. 7:**
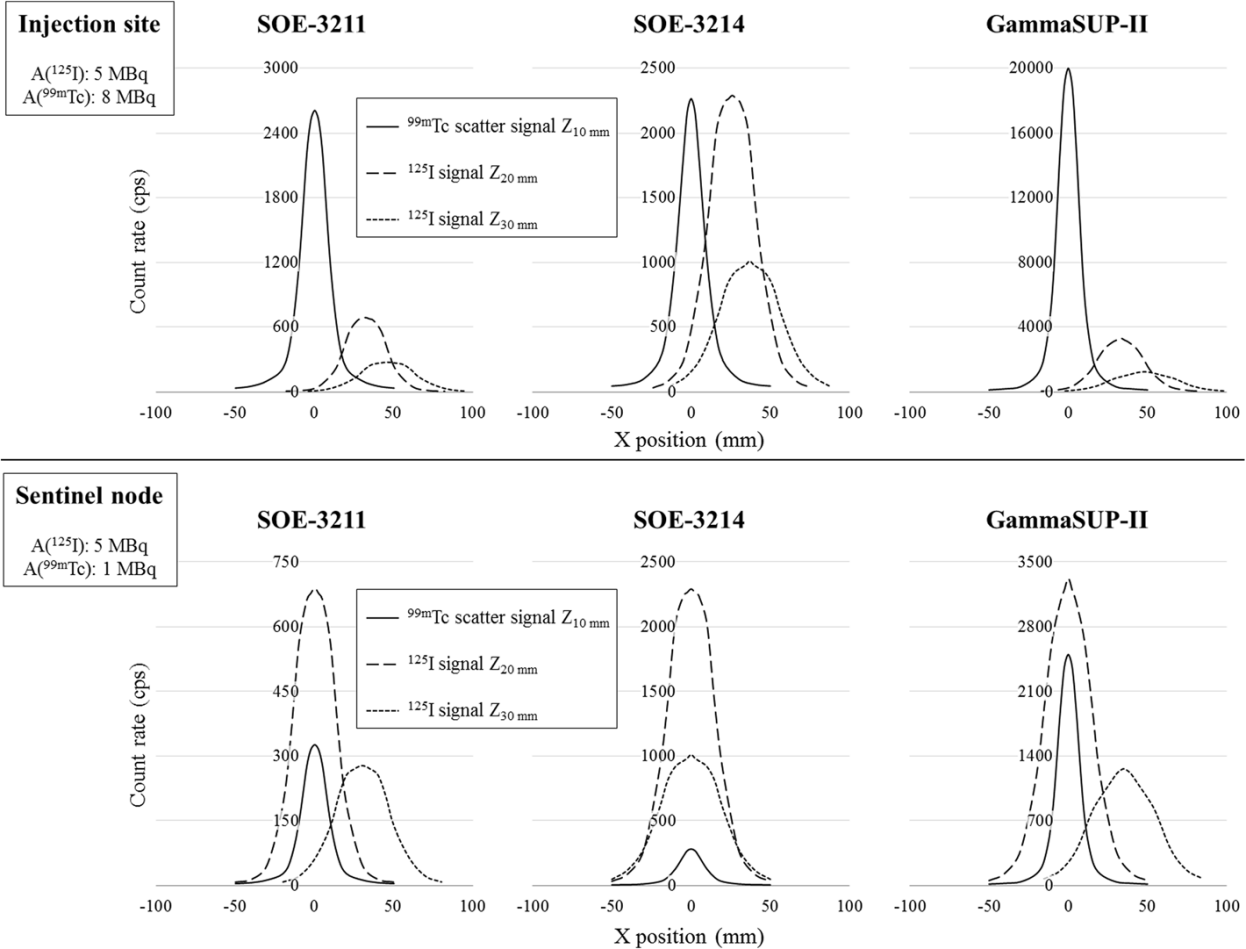
Shift Index assessments from the in vitro simulation of an 8 MBq ^99m^Tc injection site and a 1 MBq sentinel node at 10 mm depth with a 5 MBq ^125^I-seed implanted at 20 or 30 mm depth for the three probes

##### Sentinel node simulation:

The ^125^I-seed signal at 20 mm depth was higher than the ^99m^Tc scatter signal at 10 mm depth for the three probes, so no Shift Index was calculated. The ^99m^Tc scatter signal became higher than ^125^I-seed signal at 30 mm depth for the SOE-3211 and GammaSUP-II probes, with a smaller Shift Index value for the SOE-3211 probe (30 vs 35 mm).

#### ^99m^Tc scatter fraction (Fig. 8)

##### Activity ^99m^Tc scatter fraction:

The activity SF calculated from 0–1 MBq to 6.5–7.5 MBq according to the probes started from 10, 13 and 30% then decreased to 8, 10 and 12% for the SOE-3211, SOE-3214 and GammaSUP-II probes, respectively. The activity SF was linear over the whole activity range for the SOE probes and only above 5 MBq for the GammaSUP-II probe.

**Fig. 8:**
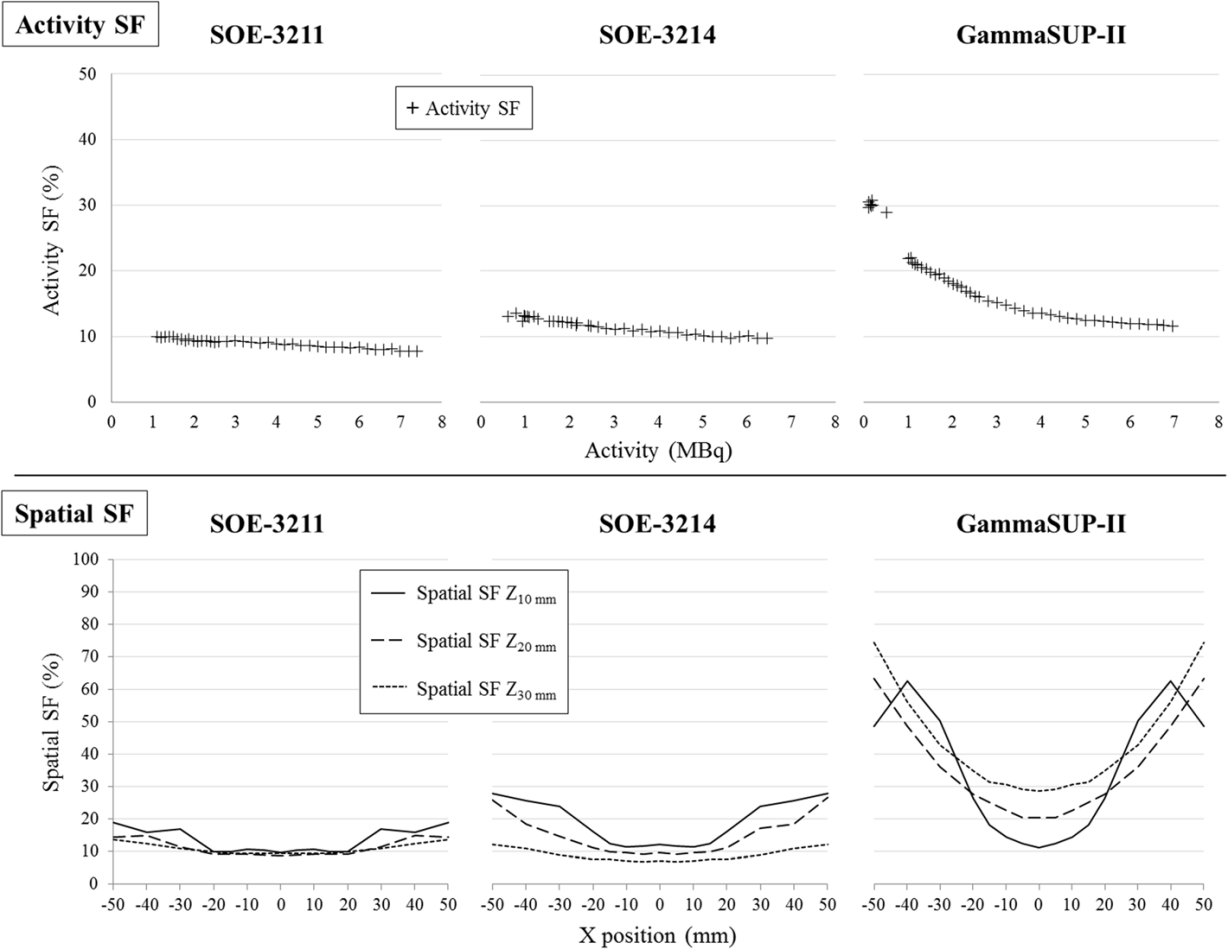
^99m^Tc scatter fractions according to the source activity and the probe positioning for the three probes

##### Spatial ^99m^Tc scatter fraction:

For the SOE-3211 and SOE-3214 probes, the spatial SF calculated at every depth was around 10% at *X*_0 mm_ and increased to 20 and 30% respectively at *X*_50 mm_. The spatial SF was higher for the GammaSUP-II probe, from 10% at (*X*_0 mm_; *Z*_10 mm_) to 70% at (*X*_50 mm_; *Z*_30 mm_).

## Discussion

Studies reporting in vivo and in vitro (based on NEMA NU3-2004 standards) probes comparisons already exist in the literature and helped us to interpret and enforce the different tests [12]. They concerned ^99m^Tc sentinel node [13] and ^18^F-guided surgery [14]. To our knowledge, such a characterisation had never been performed for ^125^I-sealed sources.

The impact of ^99m^Tc injections combined with intra-tumoural ^125^I-seed-guided surgery has already been addressed through both in vitro and in vivo studies:

- Pouw et al. confronted the performances of 3 probes simulating a ^99m^Tc intra-tumoural injection (^99m^Tc bolus and ^125^I-seed at 2 cm depth into a simulated lesion) [15].
- Hung et al. computed the ^99m^Tc downscatter contribution in the case of ipsiquadrant or periareolar injections (^99m^Tc bolus and ^125^I-seed at 1 cm depth spaced by 2 cm) and in the case of intra/peritumoural injections (^99m^Tc bolus and ^125^I-seed at the same position at 3 cm depth) using lean chicken breast [16].
- Gray et al. estimated from a mastectomy specimen, the lowest ^125^I activity needed to overcome the ^99m^Tc downscatter [17].

In every study, a non-significant impact of ^99m^Tc scatter signal was demonstrated even for the Radioguided Occult Lesion Localisation (ROLL) technique with a low activity ^125^I-seed (until 1.85 MBq).

The originality of our study was to extend the NEMA testing to calculate the Shift Index and ^99m^Tc scatter fractions. These indexes are two additional figures of merit to assess the probes performances for simultaneous nuclides detection, from the same NEMA protocol. In addition, the results from the spatial resolution test allow exhaustive in vitro simulations of any clinical configuration whatever the activity of the radioactive sources.

### Standard NEMA testing

Before performing the NEMA testing, it was essential to begin with a repeatability test to assess the warm up delay necessary to ensure a stable measurement. The count rate capability test was the first NEMA test performed in order to define the activity range involving a linear response for each measurement channel.

#### Sensitivity

The sensitivity is related to the number of photons interacting into the crystal in the selected energy window. It depends on the material (structure, density) and volume of the detection crystal as well as on the material and thickness of the entrance layer.

The GammaSUP-II probe was significantly more sensitive than the SOE probes for both ^99m^Tc and ^125^I sources despite a reduced high energy window width. The best explanation comes from the volume of its 269 mm^3^ crystal, about four times larger than the crystal of the two other probes.

The sensitivity difference between the two SOE probes with the ^99m^Tc source is also correlated with the crystal volume. Indeed, the 69 mm^3^ cubic SOE-3211 crystal is larger than the 57 mm^3^ orthocylindrical SOE-3214 crystal. Concerning ^125^I sources, the stainless-steel entrance layer of the SOE-3211 probe absorbed a part of the low energy photons, implying a loss of sensitivity compared to the SOE-3214 probe.

#### Spatial resolution in a scatter medium

The spatial resolution depends of the solid angle of detection (crystal entrance surface, probe positioning) and the discrimination of scatter signal (energy window, entrance layer) for a better focusing efficiency.

With the ^99m^Tc source, the GammaSUP-II probe allowed a better spatial resolution despite a larger entrance surface thanks to its reduced high energy window width. With the same high energy window (80–170 keV) and same crystal entrance surface (*Ø* 5 mm), the SOE probes showed logically the same spatial resolution.

The ^125^I source emitted low energy photons. Then a part of the signal, including scatter signal, was absorbed by the stainless-steel entrance layer for the SOE-3211 and GammaSUP-II probes, which improved their focusing efficiency so their spatial resolution. This phenomenon explains the fact the SOE-3211 probe had a better spatial resolution than the SOE-3214 probe despite the same crystal entrance surface (*Ø* 5 mm) and the fact the GammaSUP-II probe had the same spatial resolution as the SOE-3214 probes despite a larger crystal entrance surface (*Ø* 7 mm).

### Extended NEMA testing

#### Shift Index

The Shift Index was calculated from in vitro simulations of clinical configurations. Even if these results cannot be directly transposed into a clinical application (^99m^Tc source volume, probe oriented by a surgeon), the methodology is of interest with the definition of a new figure of merit combining spatial resolution and signal intensity for an objective assessment of signals discrimination in such a context of simultaneous sources detection.

The Shift Index results highlighted a negligible impact of the ^99m^Tc sentinel node for the ^125^I-seed detection but a potential lack of signals discrimination for a ^125^I-seed implanted below a ^99m^Tc injection site. This can be correlated to our clinical experience, indeed the only case in which the surgeon had a difficulty to find the ^125^I-seed concerned a lesion located under a ^99m^Tc injection site. Since then, it was decided to avoid ^99m^Tc injections in the breast quadrant where the ^125^I-seed is implanted.

#### ^99m^Tc scatter fraction

For the SOE probes, the activity SF was linear over the whole activity range, meaning the same saturation threshold and the same behaviour on both low and high energy windows. For the GammaSUP-II probe, the saturation threshold occurred at a higher activity in the low energy window explaining a higher and non linear activity SF below 5 MBq.

Due to attenuation and solid angle, the ^99m^Tc photopeak signal decreased according to depth (Table 3) and distance from the *X* axis origin (Fig. 5). The ^99m^Tc scatter signal, originating from both scatter medium and detector material also decreased with *X* and depth (validating our clinical simulations as the reflection of the worst detection geometry with the iodine seed aligned below the ^99m^Tc source). The spatial SF increased according to *X* for the three probes, meaning a higher relative contribution of scatter signal. However, the variation of spatial SF with depth was probe dependent (Fig. 8).

This methodology based on NEMA testing could be used by the manufacturers to improve the crystal structure and the design of the probes. In our experiments, the detector material and energy window chosen for the SOE probes allowed a reduction of the ^99m^Tc scatter fraction at lower activities and higher distances between the source and the probe.

## Conclusion

No probe stood out from the others in the whole test series; however, several observations should be pointed out:

- The three probes are suitable for distinct or simultaneous ^125^I-seed and ^99m^Tc breast cancer surgery.
- The GammaSUP-II probe allowed a better sensitivity due to its crystal volume and better spatial resolution features with the ^99m^Tc source but saturated at a lower activity.
- Considering the ^125^I-seed, the GammaSUP-II probe was more sensitive but the SOE-3211 probe showed the best spatial resolution.
- Concerning the Shift Index, the SOE-3214 probe allowed a better spatial discrimination for a ^125^I-seed implanted below a ^99m^Tc injection site or sentinel node.
- The SOE probes were more efficient while performing the ^99m^Tc scatter fraction tests.
- The SOE-3211 probe, with overall polyvalent performances, seemed to be an interesting trade-off for simultaneous detection of ^125^I and ^99m^Tc.

There is no perfect probe; the choice of the detection device has to be based on the best trade-off regarding the expected use. This in vitro study provided a complete and innovative characterisation and comparison of three different probes showing their own area of performance. Our approach based of NEMA standards allows objective and reproducible results.

## Data Availability

The datasets used and/or analysed during the current study are available from the corresponding author on reasonable request.

## Abbreviations

^125^I: Iodine 125
^99m^Tc: Technetium-99m
FWHM: Full width at half maximum
FWTM: Full width at tenth maximum
SE: Shielding effectiveness
SF: Scatter fraction index
